# Blooming Artifact Reduction in Coronary Artery Calcification by A New De-blooming Algorithm: Initial Study

**DOI:** 10.1038/s41598-018-25352-5

**Published:** 2018-05-02

**Authors:** Ping Li, Lei Xu, Lin Yang, Rui Wang, Jiang Hsieh, Zhonghua Sun, Zhanming Fan, Jonathon A. Leipsic

**Affiliations:** 1 Department of Radiology, Beijing Anzhen Hospital, Capital Medical University, Beijing, 100029 China; 2 MICT Engineering, GE Healthcare, Waukesha, WI 53188 USA; 3 Department of Medical Radiation Sciences, Curtin University, Perth, 6845 Australia; 4 Department of Radiology, St Paul’s Hospital and University of British Columbia, Vancouver, BC V6Z 1Y6 Canada

## Abstract

The aim of this study was to investigate the use of de-blooming algorithm in coronary CT angiography (CCTA) for optimal evaluation of calcified plaques. Calcified plaques were simulated on a coronary vessel phantom and a cardiac motion phantom. Two convolution kernels, standard (STND) and high-definition standard (HD STND), were used for imaging reconstruction. A dedicated de-blooming algorithm was used for imaging processing. We found a smaller bias towards measurement of stenosis using the de-blooming algorithm (STND: bias 24.6% vs 15.0%, range 10.2% to 39.0% vs 4.0% to 25.9%; HD STND: bias 17.9% vs 11.0%, range 8.9% to 30.6% vs 0.5% to 21.5%). With use of de-blooming algorithm, specificity for diagnosing significant stenosis increased from 45.8% to 75.0% (STND), from 62.5% to 83.3% (HD STND); while positive predictive value (PPV) increased from 69.8% to 83.3% (STND), from 76.9% to 88.2% (HD STND). In the patient group, reduction in calcification volume was 48.1 ± 10.3%, reduction in coronary diameter stenosis over calcified plaque was 52.4 ± 24.2%. Our results suggest that the novel de-blooming algorithm could effectively decrease the blooming artifacts caused by coronary calcified plaques, and consequently improve diagnostic accuracy of CCTA in assessing coronary stenosis.

## Introduction

Coronary CT angiography (CCTA) is a widely used non-invasive modality enabling high diagnostic performance for the diagnosis of coronary artery disease (CAD)^1–6^. Despite high diagnostic value achieved with recently developed advanced CT scanners, CCTA still has moderate specificity in the assessment of calcified plaques due to the artifacts that result from significant calcification^7^.

Calcification produces blooming and partial volume artifacts on CT imaging, which can cause erroneous enlargement of the appearance of calcification^8^. As a result blooming artifact prevents accurate evaluation of the coronary artery lumen and results in overestimation of the stenosis leading to false positive diagnosis^9–14^. Although CCTA is an excellent imaging modality for the assessment of patients with suspected CAD, calcified plaque presents a major challenge for CCTA scans.

A new vendor-specific de-blooming algorithm is under investigation to help minimize the blooming artifact of coronary calcification, and this has not been well studied. We hypothesized that this brand new algorithm would allow for more accurate evaluation of coronary artery stenosis in the presence of calcified plaques. Thus, the aim of this study was to investigate the diagnostic value of the de-blooming algorithm for evaluation of calcified plaques based on an *in vitro* phantom study and a small group of patients.

## Results

### Phantom study

#### Objective image quality on phantom study

For quantitative image quality analysis, the SNR of vessel models showed significantly higher in the group with use of the de-blooming algorithm than that of without de-blooming (STND: 52.6 ± 11.7 vs 67.9 ± 16.3, p = 0.003; HD STND: 26.8 ± 2.9 vs 33.5 ± 3.5; p = 0.005). The image noise was significantly higher in the group without use of the de-blooming algorithm (STND: 7.9 ± 2.0 vs 6.3 ± 1.9, p = 0.022; HD STND: 14.6 ± 2.8 vs 11.2 ± 2.4; p = 0.004).

#### Correlation analysis of the stenosis

**Figure 1:**
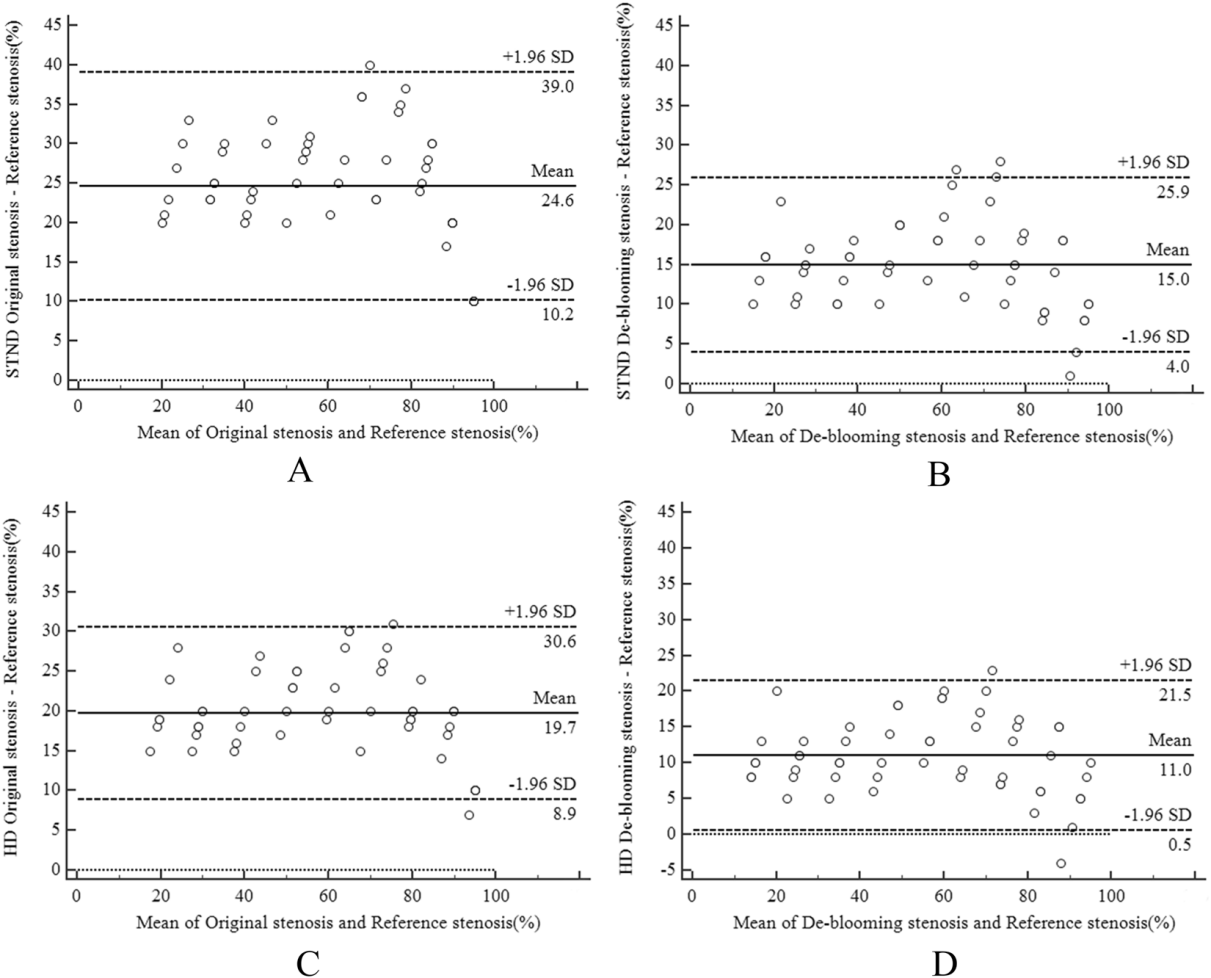
(**A**–**B**) Bland and Altman showing the correlation of Reference stenosis and Original stenosis without and with de-blooming algorithm using standard mode reconstruction (STND); (**C**–**D**) showing Reference stenosis and De-blooming stenosis without and with de-blooming algorithm using high definition mode reconstruction (HD STND). Only few cases are outside the boundary line (beyond two SD). SD = Standard deviation.

The results of the Bland–Altman comparisons are shown in Fig. 1. In the STND group, representative Bland-Altman plots of OS-RS and DS-RS showed 95% confidence limits of 10.2% to 39.0% (with a mean of 24.6%) for OS-RS, and 4.0% to 25.9% (with a mean of 15.0%) for DS-RS, respectively (Fig. 1A,B). In the HD STND group, Bland-Altman plots of OS-RS and DS-RS showed 95% confidence limits of 8.9% to 30.6% (with a mean of 19.7%) for OS-RS and 0.5% to 21.5% (with a mean of 11.0%) for DS-RS, respectively (Fig. 1C,D).

The Bland–Altman plots presented a distinct systematic overestimation of calcified plaque stenosis in CCTA, but the de-blooming stenosis is closer to reference standard than original stenosis.

#### Diagnostic performance of CCTA

**Figure 2:**
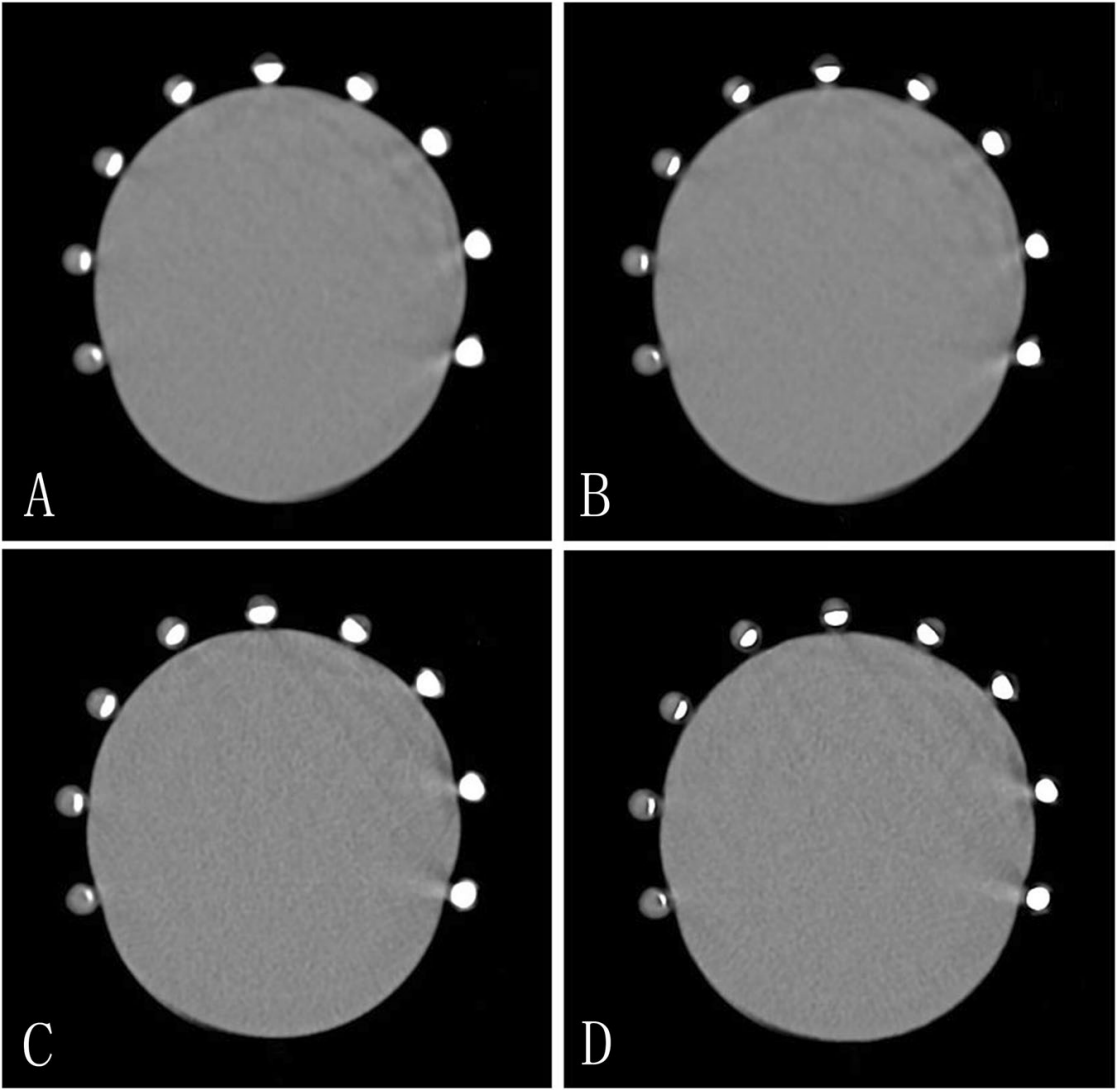
Axial CT images of the coronary vessel phantom of nine stenosis models with calcified plaques. Clockwise, in turn, is 10%, 20%, 30%, 40%, 50%, 60%, 70%, 80%, and 90% stenosis. (**A**) nine different stenoses of calcified plaques without de-blooming algorithm using standard mode reconstruction. (**B**) nine different stenoses of calcified plaques using standard mode reconstruction with de-blooming algorithm. The degree of stenosis of calcified plaques was less severe due to suppression of blooming artifacts as observed in B when compared to A. (**C**) nine different stenoses of calcified plaques using high definition standard mode reconstruction without de-blooming algorithm. The degree of stenosis of calcified plaques was less severe compared to images on A, but was more serious when compared to images on B. (**D**) nine different stenoses of calcified plaques using high definition standard mode reconstruction with de-blooming algorithm, compared with B and C, the degree of stenosis of calcified plaques was less obvious.

Figure 2 provides a representative example of the difference in calcified plaque with and without using de-blooming algorithm. Diagnostic performance of CCTA for detecting ≥50% stenosis and ≥70% stenosis is summarized in Table 1.

**Table 1:** Diagnostic Performance of CCTA in calcified plaques

	De-blooming algorithm	STND		HD STND	
		−	+	−	+
Detection of ≥50% Stenosis	Sensitivity, % (95% CI)	100(85.9–100)	100(85.9–100)	100(85.9–100)	100(85.9–100)
	Specificity, %(95% CI)	45.8(26.2–66.8)	75.0(52.9–89.4)	62.5(40.8–80.4)	83.3(61.8–94.5)
	PPV, %(95% CI)	69.8(53.7–82.3)	83.3(66.5–93.0)	76.9(60.3–88.3)	88.2(71.6–96.2)
	NPV, %(95% CI)	100(67.9–100)	100(78.1–100)	100(74.7–100)	100(80.0–100)
Detection of ≥70% Stenosis	Sensitivity, %(95% CI)	100(69.9–100)	100(69.9–100)	100(69.9–100)	100(69.9–100)
	Specificity, %(95% CI)	52.3(36.6–67.7)	64.3(48.0–78.0)	59.5(43.4–74.0)	76.2(60.2–87.4)
	PPV, %(95% CI)	37.5(21.7–56.3)	44.4(26.0–64.4)	41.4(24.1–60.9)	54.6(32.7–74.9)
	NPV, %(95% CI)	100(81.5–100)	100(84.5–100)	100(83.4–100)	100(86.7–100)

PPV, positive predictive value; NPV, negative predictive value; CI, confidence interval; STND: standard mode reconstruction; HD STND: high definition mode reconstruction.

For detecting ≥50% stenosis, when recon type is STND, specificity increased from 45.8% (95% confidence interval [CI] 26.2–66.8) to 75.0% (95% CI 52.9–89.4) for the detection of 50% or greater stenosis with the use of de-blooming algorithm; while PPV increased from 69.8% (95% CI 53.7–82.3) to 83.3% (95% CI 66.5–93.0). When recon type is HD STND, specificity increased from 62.5% (95% CI 40.8–80.4) to 83.3% (95% CI 61.8–94.5) for detection of 50% or greater stenosis with the use of de-blooming algorithm; while PPV increased from 76.9% (95% CI 60.3–88.3) to 88.2% (95% CI 71.6–96.2).

For detecting ≥70% stenosis, when recon type is STND, specificity increased from 52.3% (95% CI 36.6–67.7) to 64.3% (95% CI 48.0–78.0) for the detection of 70% or greater stenosis with the use of de-blooming algorithm; while PPV increased from 37.5% (95% CI 21.7–56.3) to 44.4% (95% CI 26.0–64.4). When recon type is HD STND, specificity increased from 59.5% (95% CI 43.4–74.0) to 76.2% (95% CI 60.2–87.4) for detection of 70% or greater stenosis with the use of de-blooming algorithm; while PPV increased from 41.4% (95% CI 24.1–60.9) to 54.6% (95% CI 32.7–74.9).

The specificity and PPV were significantly higher in the two groups after using de-blooming algorithm; and the specificity and PPV were significantly higher in the HD group than that in the STND group. Overall, diagnostic accuracy of CCTA was significantly improved with the use of the de-blooming algorithm.

### Patient study

The study population consisted of 31 patients with a mean age of 63.4 ± 5.0 years. Twenty-two (71.0%) patients were male. The mean HR during the scan was 70.5 ± 8.8 bpm. The mean body mass index was 24.5 ± 2.5 (18.8–32.7) kg/m^2^. The mean radiation dose was 1.4 ± 0.7 mSv.

#### Subjective image quality assessment

A total of 375 coronary artery segments from 31 patients were included for evaluation; image quality was comparable with and without use of de-blooming algorithm. Without its use, 92.8% (348/375) of the segments were rated as diagnostic (score 1–3). For all of the coronary segments, 26.7% (100/375) had excellent image quality (score 1); 46.4% (174/375) had good image quality (score 2); 19.7% (74/375) had adequate image quality (score 3); and 7.2% (27/375) were of non-diagnostic image quality (score 4).

With use of de-blooming algorithm, 98.9% (371/375) of the segments were rated as diagnostic (score 1–3). For all of the coronary segments, 31.7% (119/375) had excellent image quality (score 1); 52.8% (198/375) had good image quality (score 2); 14.4% (54/375) had adequate image quality (score 3); and 1.1% (4/375) were of non-diagnostic image quality (score 4).

#### Quantitative assessment of reduction of plaque calcification, plaque volume and diameter over calcified plaque (%)

A total of 77 plaques were analyzed in the left anterior descending (n = 32), the left circumflex (n = 12), the right coronary artery (n = 14), the left main coronary artery (n = 6), the diagonal branch (n = 9), the ramus (n = 2), the obtuse marginal branch (n = 1) and the posterior descending branch (n = 1). In 3 plaques, the edge-detection algorithm of the software tool failed to correctly identify the plaque boundaries. Therefore, the final analysis included 74 plaques in 29 patients.

**Figure 3:**
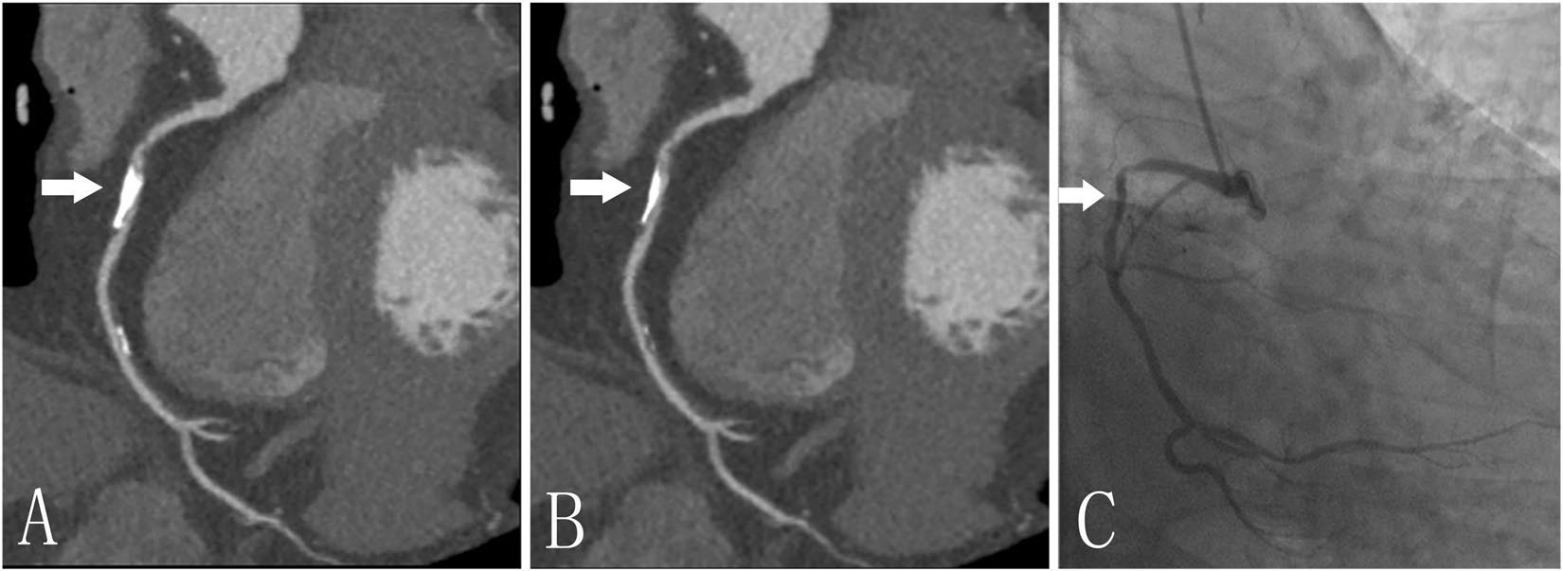
A 77-year-old man with calcified plaque (arrow) of the right coronary artery. A and B, Curved planar reformation (CPR) images without (**A**) and with (**B**) de-blooming algorithm show the volume reduction of calcified plaque and improvement of lumen evaluation by using this algorithm; (**C**) Invasive coronary angiography confirms mild stenosis in the right coronary artery. Without de-blooming algorithm, the right coronary artery was almost occluded; while with use of de-blooming algorithm, the right coronary artery was almost 50% stenosis, being consistent\ with findings from invasive coronary angiography.

The volume of calcified plaques with the de-blooming algorithm decreased from 35.1 ± 26.5 mm^3^ to 19.3 ± 15.8 mm^3^ (p < 0.0001); While coronary diameter stenosis decreased from 34.7 ± 21.4% to 18.4 ± 15.4% (p < 0.0001); coronary area stenosis decreased from 50.1 ± 24.6% to 27.2 ± 19.8% (p < 0.0001). The reduction of calcification volume was 48.1 ± 10.3%, while reduction of coronary diameter stenosis was 52.4 ± 24.2%; and coronary area stenosis reduction over calcified plaque was 51.1 ± 23.3%. Figure 3 provides a representative example with and without use of de-blooming algorithm.

## Discussion

Our results from phantom experiment and patient study suggest that the new de-blooming algorithm appears to effectively decrease the blooming artifacts caused by coronary calcified plaques, and improve image interpretability and diagnostic accuracy. Further, we also found a trend in our analysis that HD reconstruction mode improved coronary artery luminal visualization and thus results in higher diagnostic accuracy in comparison with standard reconstructions mode.

During CCTA scans, calcified plaques cause two types of artifacts. One is blooming artifact and the other is beam-hardening or streak artifact. Blooming artifact is caused by limited spatial resolution, which is associated with the design tradeoffs between image noise and resolution, and with the partial volume averaging of different densities within a single voxel. High-density calcification contaminates the density of other tissues in the voxel and adjacent voxels, and exaggerates the size of the calcified plaque^15^. The high-density calcified plaques appears larger than the actual size since the presence of blooming artifact, which prevents accurate evaluation of the coronary artery lumen, and results in overestimation of the severity of coronary stenosis as shown in our phantom study. This may result in a false positive diagnosis of CCTA in clinical setting, and reduce its specificity, which may lead to additional downstream testing, including ICA^10,11,16,17^.

There have been some solutions to reduce blooming artifacts from scanning and data post-processing. Increase of the spatial resolution using thinner collimation and reconstruction thicknesses, as well as higher-resolution, sharper reconstruction algorithms can reduce blooming artifacts with the expense of higher image noise even at higher radiation dose^15^. A CCTA study compared high spatial resolution scanning (0.23 mm) with standard scanning (0.625 mm), and found improved diagnostic accuracy in the evaluation of coronary calcified plaques (fewer false positive results)^18^. The spatial resolution along the z-axis of CT scanner is mainly based on the slice thickness during data acquisition^19^. However, the detector size or slice thicknesses of the latest-generation CT scanners has not improved since the 64-row CT, ranging from 0.5 to 0.625 mm for different vendors. High-convolution filter reconstruction algorithms for post-processing the acquired images may have beneficial effects on calcium visualization^20^. Implementation of image post-processing algorithms has been shown to reduce blooming artifacts to some extent^21^. Studies have compared iterative reconstruction (IR) with filtered back projection (FBP) reconstruction techniques for the evaluation of heavily calcified vessels, the results demonstrated that IR improved the per-segment diagnostic accuracy compared to FBP, since IR reduces image noise and potentially reduce blooming artifacts^21,22^. Increase of the scanning kVp was proposed previously. However, high kVp only reduces beam-hardening artifacts, blooming artifacts are not affected because the spatial resolution is unchanged^23^. More recently, novel technologies for removal of calcium from CTA images have been reported. The feasibility of calcium subtraction in carotid arteries using dual-energy CT as well as its accuracy for stenosis assessment compared to invasive angiography has been described^24–26^. However, there are currently no reports of calcified plaque subtraction in coronary artery using dual-energy CT based on patient studies^27^.

All solutions listed above have limited impact on the blooming artifact reduction in coronary calcification. To overcome these limitations, we adopted a brand new de-blooming algorithm. Main factors that contribute to the cardiac blooming artifact are beam-hardening effect, finite spatial resolution of the CT system, and patient motion. The first two effects are deterministic in the sense that these factors can be modeled and predicted prior to the patient scan. The patient motion induced blooming artifacts, on the other hand, are quite complex and need to be addressed on a case-by-case basis. The de-blooming algorithm under investigation in this paper focuses on addressing the first two effects: beam-hardening and finite spatial resolution. The algorithm first models the blooming effect based on the known x-ray spectrum used in the scan as well as the convolution kernel used for the reconstruction. Based on the modeling, point-spread-functions (PSF) of the system under different conditions are generated. The goal of the algorithm is to deconvolve the corresponding PSF and restore the underline signal affected by the high-density objects, such as calcified plaques, for the particular scan. Because of the complexity of the deconvolution process, the algorithm is iterative in nature. It tries to minimize the difference between the reconstructed image and the corrected image convolved with the PSF. The iterative approach overcomes many of the shortcomings, such as overshoot or undershoot, of the traditional deconvolution approaches^28^.

The present study showed that this de-blooming algorithm could significantly reduce the effect of blooming. The measured stenosis of coronary calcification is close to the ground truth after using this de-blooming algorithm. The image interpretability and diagnostic accuracy was significantly improved. Thus, this preliminary study shows the potential value of this new algorithm for improving diagnostic assessment of calcified plaques, although findings need to be confirmed by a larger cohort of patients.

Since the appearance oversizing of calcium can be reduced by increasing display window width and window level during interpreting CCTA images, such approach has the potential to reduce blooming artifact^29,30^. The recommended window width/window level for calcified plaques is 1500/300 HU^29^. This window setting was used for all coronary calcification analysis in our study. In our phantom study, there was “dark rim” artifact on the edge of the simulated calcified plaque. We consider that this phenomenon was caused by beam hardening artifact due to the high density of calcification. However, this dark rim artifact was not obvious in the preliminary patient study of coronary calcified plaques.

Several limitations in this study have to be addressed. Firstly, the simulated calcified plaque used to detect the de-blooming effect in the phantom study is obviously different from the calcified plaque in the patients^31–33^. Nevertheless, the effect of this de-blooming algorithm in preliminary patient study is very promising. Secondly, although our results demonstrated that the use of this de-blooming algorithm significantly improves the stenosis evaluation in calcium at 60 bpm, further studies are needed to assess the effect of this new algorithm at higher heart rates. Thirdly, although those measurements were from auto-analysis plaque software, there are potential biases due to the default settings of the software. Fourthly, the vessel phantoms with only inner diameter of 3.5 and 4 mm were investigated, while the simulated vessels with diameter of less than 3.5 mm were not studied. Fifthly, The algorithm used in this study is vendor specific and is limited to CT scanner produced by GE Healthcare (Revolution CT, GE healthcare, Waukesha, WI) only. Finally, the sample size of the preliminary patient study is very small due to the fact that this new algorithm was only recently introduced in our practice. Further research on the diagnostic performance of CCTA using this new algorithm in calcified plaque with comparison of ICA as the gold standard for the diagnosis of stenosis is warranted.

In conclusion, our results showed that the use of this new de-blooming algorithm significantly reduces the blooming artifact in coronary calcified plaques. This technique can improve image interpretability, and diagnostic accuracy of CCTA for evaluation of significant stenosis in the presence of calcified plaques. This technique has the potential to overcome the current challenges of CCTA for overestimation of stenosis severity in calcified plaques due to blooming artifacts. It will bring new opportunities for accurate plaque assessment on CCTA. Further studies with inclusion of large cohort of patients are required to verify our findings before the new de-blooming algorithm is widely recommended in clinical practice.

## Methods

### Phantom study

#### Calcified coronary artery phantom

Coronary arteries were simulated using vessel phantoms with inner diameter of 3.5 and 4 mm. The vessel phantoms were constructed to include three relevant components of calcified arteries, including blood pool, vessel wall, and calcification. The vessel wall was formed from polymethyl methacrylate with a wall thickness of 1.0 mm. A total of 54 calcified plaques with different sizes and stenoses were simulated. Calcifications were mainly composed of hydroxyapatite with CT value of 1097–2910 Hounsfield Units (HU) at 100 kVp. The shape of the calcifications was regular, and they were placed inside the vessel phantoms. The vessel phantom consisted of nine stenosis models with calcified plaques. Stenoses of 10%, 20%, 30%, 40%, 50%, 60%, 70%, 80%, and 90% were simulated. For a vessel diameter of 4.0 mm, the plaque sizes were created as 0.4 mm, 0.8 mm, 1.2 mm, 1.6 mm, 2.0 mm, 2.4 mm, 2.8 mm, 3.2 mm, and 3.6 mm to correspond to 10% to 90% stenosis.

#### Original cardiac phantom

A pulsating cardiac phantom (FYC FUYO Corporation, Tokyo, Japan) which contained a silicone chamber to simulate the left ventricle (LV) was used. The chamber was directly connected to a cardiac driver pump controlled by a computer program that simulated ECG signals and determined heart rate of the model, which allowed performing ECG-gated CCTA. The LV model was pulsated by the pump with simulated heart rate (HR) of 60 beats per minute (bpm). The vessel models were attached to the surface of the LV model.

The models of the LV and coronary vessels were filled with diluted contrast medium (370 mg iodine/ml, iopromide; Ultravist, Bayer Schering Pharma, Berlin, Germany). The CT attenuation value of diluted contrast medium was 335–365 HU at 100 kVp. The long axis of the LV and vessel models was arranged parallel to the z-axis of the CT gantry.

#### CCTA

CCTA was performed using a 256-row detector CT scanner (Revolution CT, GE healthcare, Waukesha, WI). A scout image was acquired to determine the scan range to cover the entire LV and vessel models. CCTA was then performed using a prospectively ECG-triggering volume scan protocol with standard-resolution scanning mode and the high-resolution scanning mode, respectively. The exposure window was set at 0–100% of the R-R interval. Scanning parameters were as follows: tube voltage, 100 kVp; tube current, 350 mA; temporal resolution, 140 milliseconds; display field of view (DFOV), 12 cm; scan range, 80 mm; and collimation, 256 × 0.625 mm. The CCTA scans were performed at HR of 60 bpm.

#### Image postprocessing

After CCTA scanning, multiphase cardiac axial images were reconstructed with 0.625 mm slice thickness and 0.625 mm interval using an iterative reconstruction-V algorithm(ASIR-V, GE Healthcare) at 50% strength. Optimal cardiac phase with the least motion artifact was selected manually by the operator. Two different reconstruction kernels, standard (STND) and high definition (HD STND),were used for standard- and high-resolution modes of scans, respectively. The DICOM data of reconstructed images were transferred to an off-site computer for de-blooming processing. The data before and after the de-blooming processing were analyzed using a dedicated workstation (Advantage Workstation 4.6; GE Healthcare).

#### Image analysis

An experienced reader (R1, cardiovascular radiologist with 8 years of experience in cardiovascular radiology) independently reviewed all data sets of the coronary vessel models with the same display window parameters (width, 1500; level, 300)^29^. The datasets consisted of images with four different reconstruction and processing methods and with various degree of stenosis (10–90%) each. These four different methods included standard (STND) with and without de-blooming algorithm and HD-standard (HD STND) with and without de-blooming algorithm. The reader was asked to measure maximal stenosis severity using auto-analysis plaque software in dedicated workstation (Advantage Workstation 4.6; GE Healthcare).

For image quality analysis, the CT attenuation was measured within the vessel segment without calcium and the periphery outside the vessel phantom. The standard deviation of the CT attenuation value at the periphery was measured as image noise. Signal-to-noise ratio (SNR) was calculated for each measurements as follows: SNR _vessels_ = CT_vessels_/SD_periphery_.

### Patient study

#### Population

Ethics approval was received from the Ethics Committee of Beijing Anzhen Hospital. The informed consent was waived by the institutional review board due to the purely retrospective nature of this study. This study was performed following the Declaration of Helsinki which was revised in 2008.

This retrospective study included 31 patients who were referred for the CCTA assessment of known or suspected CAD. Patients with at least one coronary artery segment with a calcified plaque were included. Patients with non-calcified plaque, with coronary artery bypass grafting and coronary stenting were excluded from this study group.

#### CT acquisition and postprocessing

All patients underwent CCTA using the same CT scanner with prospectively ECG-triggering volume scan within a single cardiac cycle. Automatically selected tube voltage by kV-Assist and tube current by Smart-mA was set according to the scout image. The data acquisition window was set at 35–80% of the R-R interval according to HR. After placing an 18-G intravenous catheter through an antecubital vein, contrast medium of 60–65 ml (370 mg iodine/ml, iopromide; Ultravist, Bayer Schering Pharma, Berlin, Germany) was injected at 4–5 ml/s rate followed by 30 ml saline with a dual-head power injector. Scanning parameters included 256 × 0.625 mm collimation, and scan coverage was 120 or 140 mm with a matrix size of 512 × 512 pixels and reconstruction section thickness and section interval of 0.625 mm.

Images were reconstructed at the optimal cardiac phase using the iterative reconstruction algorithm at 50% strength (ASIR-V) with standard convolution kernel. Motion correction technology (Snap Shot Freeze, SSF) was used when motion artifact was present. The reconstructed axial CCTA images were transferred to an off-site computer for de-blooming processing.

#### Image analysis

Two experienced radiologists (R2, cardiovascular radiologist with 10 years of experience in cardiovascular radiology; R3, cardiovascular radiologist with 12 years of experience in cardiovascular radiology) reviewed the image quality of CCTA with and without de-blooming algorithm with consensus approach using an 18-segment model^22^. Image quality was rated using a 4-point scale. 1 = excellent image quality free of artifacts; 2 = good image quality with minor artifacts, but fully evaluable and diagnostic; 3 = adequate image quality with moderate artifacts, but acceptable for diagnosis; 4 = poor/severe artifacts and non-diagnostic image quality.

One experienced reader independently reviewed all data sets, noted coronary calcification, and measured the volume of calcified plaques, coronary diameter stenosis(%), and coronary area stenosis(%) using a commercially available software Autoplaque (AUTOPLAQ; Cedars-Sinai Medical Center, Los Angeles, CA). The same display window parameters (width, 1500; level, 300) were applied for all imaging review. The reduction of calcification volume (RCV), coronary diameter stenosis reduction (RDS), and coronary area stenosis reduction over calcified plaque (RAS) were calculated as follows:

$$\begin{array}{c}{\rm{RCV}}=({{\rm{CV}}}_{{\rm{without}}{\rm{deblooming}}}-{{\rm{CV}}}_{{\rm{with}}{\rm{deblooming}}})/{{\rm{CV}}}_{{\rm{without}}{\rm{deblooming}}}\\ {\rm{RDS}}=({{\rm{DS}}}_{{\rm{without}}{\rm{deblooming}}}-{{\rm{DS}}}_{{\rm{with}}{\rm{deblooming}}})/{{\rm{DS}}}_{{\rm{without}}{\rm{deblooming}}}\\ {\rm{RAS}}=({{\rm{AS}}}_{{\rm{without}}{\rm{deblooming}}}-{{\rm{AS}}}_{{\rm{with}}{\rm{deblooming}}})/{{\rm{AS}}}_{{\rm{without}}{\rm{deblooming}}}\end{array}$$

### Statistical analysis

All continuous variables were expressed as mean ± SD. For the statistical analysis, MedCalc (MedCalc Software, version 15; Ostend, Belgium) and SPSS software (SPSS, version 20.0; IBM Corporation, Armonk, NY, USA) were used. For phantom study, Bland-Altman plots were performed to analyze the correlation of reference standard (RS) with original stenosis (OS) and de-blooming stenosis (DS). The sensitivity, specificity, positive predictive value (PPV), and negative predictive value (NPV) of CCTA for detection of ≥50% stenosis and ≥70% stenosis with and without using de-blooming algorithm were calculated with the actual stenosis as reference standard. For patient study, the volume of calcified plaques (mm^3^), coronary diameter stenosis (%) and coronary area stenosis over calcified plaque (%) with and without using de-blooming algorithm were compared. P < 0.05 was considered statistically significant.

### Data availability

All data generated or analysed during this study are included in this published article. Additional datasets are available from the corresponding author on reasonable request.
